# Genome Sequence of Complex HIV-1 Unique Recombinant Forms Sharing a Common Recombination Breakpoint Identified in Malaysia

**DOI:** 10.1128/genomeA.01220-15

**Published:** 2015-11-05

**Authors:** Hui Ting Cheong, Kim Tien Ng, Lai Yee Ong, Yutaka Takebe, Kok Gan Chan, Clayton Koh, Haider Abdulrazzaq Abed Al-Darraji, Adeeba Kamarulzaman, Kok Keng Tee

**Affiliations:** a Centre of Excellence for Research in AIDS (CERiA), Department of Medicine, Faculty of Medicine, University of Malaya, Kuala Lumpur, Malaysia; b AIDS Research Center, National Institute of Infectious Diseases, Tokyo, Japan; c School of Medicine, Yokohama City University, Yokohama, Kanagawa, Japan; d Division of Genetics and Molecular Biology, Institute of Biological Sciences, Faculty of Science, University of Malaya, Kuala Lumpur, Malaysia

## Abstract

Three strains of HIV-1 unique recombinant forms (URFs) descended from subtypes B, B′, and CRF01_AE were identified among people who inject drugs in Kuala Lumpur, Malaysia. These three URFs shared a common recombination breakpoint in the reverse transcriptase region, indicating frequent linkage within the drug-injecting networks in Malaysia.

## GENOME ANNOUNCEMENT

Error-prone reverse transcription, high viral turnover rates, and tendencies to undergo genetic recombination in the human immunodeficiency virus type 1 (HIV-1) have generated an immense number of variants circulating worldwide (1). As of 2015, more than 70 circulating recombinant forms (CRFs) have been identified globally (http://www.hiv.lanl.gov/). In Malaysia, various HIV-1 CRFs such as CRF33_01B, CRF48_01B, CRF52_01B, CRF53_01B, CRF54_01B, CRF58_01B, and CRF74_01B (2–8) have been described in the past decade. In this study, we determined three near-full length genomes of unique recombinant forms (URFs), designated 08MYKL056, 10MYKJ086, and 10MYPR70, and found that they share one unique recombination breakpoint in the genome.

HIV-1 RNA was extracted using the NucliSENS easyMAG automated platform (bioMérieux, France). HIV-1 strains from three epidemiologically unrelated males were subjected to near-full length PCR amplification using the overlapping PCR approach (2). PCR products were purified and sequenced in an ABI PRISM 3730XL DNA analyzer with BigDye terminators (Applied Biosystems, Foster City, California, USA). Nucleotide sequences generated were then assembled and aligned prior to recombination and phylogenetic analyses (similarity plot, bootscanning, informative sites analyses, and neighbor-joining). The recombination analyses for all three genomes (spanning the *gag, pol, env, rev, vif, vpr, vpu*, and *nef* genes) were carried out using CRF01_90THCM240 (accession number U54771) and subtype B′ _93CNRL42 (accession number U71182) as putative parental subtypes and subtype C_95IN21068 (accession number AF067155) as an outlier.

The first strain 08MYKL056 (HXB2: 709 to 9,605; 8,915 bp) with thirteen recombination breakpoints was identified from a 35-year-old male patient with unknown risk factors. A total of fourteen subgenomic regions alternating between CRF01_AE subtype B and subtype B′ were identified. Seven CRF01_AE subgenomic regions were in HXB2 positions 1,839 to 1,941, 2,040 to 2,179, 2,846 to 3,284, 3,758 to 5,839, 5,987 to 6,124, 6,223 to 8,446, and 9,463 to 9,605, and seven subtype B (western origin) or B′ (Thai origin) regions were in HXB2 positions 709 to 1,816 (B′), 1,942 to 1,971 (B or B′), 2,202 to 2,789 (B), 3,317 to 3,747 (B′), 5,860 to 5,945 (B′), 6,125 to 6,222 (B or B′), and 8,498 to 9,436 (B). The second strain 10MYKJ086 (HXB2: 761 to 8,818; 8,064 bp) was identified from a male patient with the history of injecting drug use. Two CRF01_AE subgenomic regions were in HXB2 positions 2,876 to 5,519 and 5,988 to 8,818, while two subtype B′ subgenomic segments were observed in HXB2 positions 761 to 2,840 and 5,582 to 5,945. The third strain 10MYPR70 (HXB2: 742 to 8,835; 8,062 bp) with two recombination breakpoints was identified from a 46-year-old male patient who reported prior injecting drug use. Three subgenomic regions of CRF01_AE, subtype B′ and CRF01_AE were identified at HXB2 positions 742 to 2,052, 2,064 to 2,840, and 2,876 to 8,835, respectively.

We noted that strains 08MYKL056, 10MYKJ086, and 10MYPR70 shared a recombination breakpoint between subtype B′ and CRF01_AE in the reverse transcriptase region at HXB2 positions 2,841 to 2,845, suggesting that these three strains were arisen from a common ancestral subtype B′/CRF01_AE recombinant strain through complex drug injecting networks in Malaysia.

### Nucleotide sequence accession numbers.

The genome sequences of strains 08MYKL056, 10MYKJ086, and 10MYPR70 have been deposited in GenBank under the accession numbers KT438782, KT438783, and KT438784, respectively.
